# Perceptions and intervention preferences of Moroccan adolescents, parents, and teachers regarding risks and protective factors for risky sexual behaviors leading to sexually transmitted infections in adolescents: qualitative findings

**DOI:** 10.1186/s12978-019-0801-y

**Published:** 2019-09-10

**Authors:** Hicham El Kazdouh, Abdelghaffar El-Ammari, Siham Bouftini, Samira El Fakir, Youness El Achhab

**Affiliations:** 1 Laboratory of Epidemiology, Clinical Research and Community Health, Faculty of Medicine and Pharmacy of Fez, B.P 1893, Km 2.2 Route Sidi Harazem, 30000 Fez, Morocco; 2 Regional Centre for Careers Education and Training of Fez-Meknes, Fez, Morocco

**Keywords:** Adolescents, Morocco, Sexual health, Qualitative study, Intervention, Risk and protective factors, Sexually transmitted infections, Socio-ecological model, School

## Abstract

**Background:**

Sexual choices and practices of adolescents living in conservative societies, including Morocco, can be influenced either positively or negatively by the prevailing contextual and social norms. These norms not only limit the access to reproductive health information and services but also lead to abstinence among devout adolescents. Thus, identifying contextual risks and protective factors of risky sexual behaviors leading to sexually transmitted infections (STIs) in adolescents, as well as exploring perceptions of adolescents, parents and teachers regarding effective intervention preferences could improve the sexual health of adolescents.

**Methods:**

We conducted a qualitative study using focus group discussions (FGDs) based on the socio-ecological model as a theoretical framework. Sample groups of adolescents, parents, and teachers were selected from two public middle schools (disadvantaged and advantaged according to socio-economic level) in Taza city, Morocco, from May to July 2016. Participants were polled on protective factors and perceived facilitators of risky sexual behaviors leading to sexually transmitted infections (STIs) in adolescents as well on their perception of intervention preferences to reduce the risks. Three sets of data were initially formed, coded, and analyzed using thematic analysis.

**Results:**

Seventeen FGDs were conducted, including 8 groups of adolescents (28 boys and 28 girls, 14–16 years old), 5 groups of parents (21 males and 5 females), and 4 groups of teachers (13 males and 5 females). Five overall themes seemed to influence risky sexual behaviors in adolescents: (1) risky sexual practices and STIs; (2) the adolescent’s social domain; (3) the role of school; (4) media, including internet and social media; and (5) socio-cultural norms. Participants also suggested a number of possible interventions to improve the sexual health of adolescents and to reduce the risk of STIs, which could be applied at multiple levels.

**Conclusions:**

Successful intervention programs should target the multifaceted factors affecting the adolescent’s sexual behaviors, from the individual to the societal level. Allowing parents, teachers, and adolescents to work together could help reduce the socio-cultural and personal barriers that prevent effective communication about sexuality. Furthermore, schools can play a vital role in reducing risky sexual behaviors and STI acquisition rates in adolescents by promoting sex education in school curriculum and encouraging adolescents to engage in extracurricular activities and awareness campaigns.

## Plain English summary

We conducted a qualitative study using focus group discussions to identify the perceptions of adolescents, parents, and teachers regarding factors that affect adolescents’ sexual behavior that make them vulnerable to sexually transmitted infections (STIs), and those that protect adolescents by helping them to make healthy choices regarding reproductive health. In addition, participants were asked about their perceptions of intervention preferences. We identified five overall themes that seemed to influence sexual behavior in adolescents: (1) risky sexual practices and STIs; (2) the adolescent’s social domain; (3) the role of school; (4) media, including internet and social media; and (5) socio-cultural norms. Participants also proposed a number of programs to improve adolescent sexual health and reduce the risk of STIs, which could be multileveled. Crucially, implementing an effective sexual health intervention is contingent on the multilateral contributions of parents, teachers, and officials. Programs to improve sexual and reproductive health in adolescents should consider the various identified factors affecting adolescent sexual behavior and the identified suggestions for preferred interventions.

## Introduction

Globally, there were 36.7 million people living with human immunodeficiency virus (HIV) in 2016. Of these, 2.1 million were adolescents; 770,000 younger (age 10–14 years) and 1.03 million older (age 15–19 years) [1, 2]. In addition, the prevalence of sexually transmitted infections (STIs) remains high, more than 357 million new curable infections (such as chlamydia, gonorrhea, trichomoniasis, and syphilis) were estimated in 2012 [3]. The highest rates of STIs were observed among 15–24 years old, followed by 15–19 years old [4]. Despite available treatments, STIs account for high morbidity, mortality and other complications such as infertility, psychosocial problems, and economic productivity losses [5].

In developing countries, including the Middle East and North Africa (MENA), several studies have indicated the rising prevalence of STIs among adolescents and youth [4, 6, 7]. This risk seems to exacerbate by the lack of awareness and limited access to reproductive health services [8–12]. About 400,000 new cases of STIs are recorded throughout public health clinics in Morocco each year [13]; 40% of these cases are between 15 and 29 years [14]. According to UNAIDS estimates, there were 22,000 patients with HIV in Morocco in 2016, less thant 500 of whom were children (0–14 years) [15]. Additionally, 4800 adolescents (10–19 years) were living with HIV in MENA countries, including Morocco in 2017. However, the epidemic trend in this region remains uncertain, due to the lack of critical data in 14 out of 19 countries [16]. The estimated number of children and adolescents with HIV in Morocco was less than 1000, similar to Algeria and lower than Egypt with 1400 infected adolescents [16]. It is assumed that adolescents are completely aware of STIs risks and preventive factors thanks to the role of school, especially in conservative countries where religiosity is a strong protective factor against risky behaviors [17–19]. Nevertheless, several studies indicated that many adolescents in Arab countries, including Morocco, are still unaware of risky sexual behaviors and STIs [8, 9, 11, 19–26].

There is evidence that sexually active adolescents are at an increased risk for STIs, including HIV, compared with other age groups because of behavioral, biological, and cultural reasons combined [2, 4, 6, 10, 27]. Adolescence is a period of life in which teens reach sexual maturity, and begin to make reproductive health decisions and choices based on their background knowledge and the availability of reproductive health services, especially with regard to abstinence, use of condoms and contraceptives, and decisions to keep a pregnancy [11, 28, 29]. During this age period, many adolescents engage in their first sexual practices that are prone to reproductive health problems lasting into adulthood [10, 28, 30, 31]. It is also a period of exploration and experimentation, more susceptible to STIs. Adolescents are also more vulnerable because of their lack of knowledge about STIs and their involvement in risky sexual practices, including early sexual initiation, unsafe sex, multiple sexual partners, and inconsistent condom use [12, 19, 22, 23, 28, 31–34]. Concurrently, adolescence is also a formative period, where positive behaviors and healthy lifestyles can be learned and established [20, 35]. Arguably, interventions that promote adolescent health and that provide relevant information about STIs and facilitate access to adolescent health services have been proven to have positive effects on the reproductive health of adolescents [34, 36, 37].

The decision to engage in risky sexual behaviors has been shown to be influenced by a number of facilitating and protective factors [28, 38, 39]. Globally, a number of studies have already been conducted to determine risks and protective factors associated with sexual activity among school-going adolescents [11, 22, 39–41]. These and others have shown that poor knowledge about sexuality and STIs, substance use, peer pressure, adolescent curiosity, poverty, poor parental supervision, and globalization are strongly associated with adolescent risky sexual activity [25, 28, 31, 33, 38–40, 42]. Yet, religiosity, having close friends, peer support, parental supervision, parental connectedness, parental bonding, and formal and parental sex education, including abstinence, can play a protective role to decrease risky sexual behaviors [17, 18, 27, 38, 40].

There is continuing need of identifying factors associated with risky sexual activity and STI acquisition among adolescents to better design effective intervention programs. Factors that protect or promote risky sexual behaviors in adolescents can be found at many levels, from the individual to the societal at large [32]. Consequently, the socio-ecological model offers a convenient organizing framework to explore how multiple factors that interact at various levels may increase or decrease risky sexual behaviors among adolescents, which could then be used to guide successful health promotion interventions [32, 43]. The principle of this theoretical framework is that the social context appears to have an important influence on the sexual choices and reproductive health behaviors of adolescents, which should be included in approaches to promote health behaviors [32, 43]. In the socio-ecological model [43], the factors underlying risky sexual behaviors among adolescents have been categorized into four levels: individual, relationship, community, and societal.

So far, studies on adolescent sexual health in developing countries have been concentrated in sub-Saharan African countries and have focused mainly on individual- and family-level factors that influence reproductive health decisions and choices among adolescents; factors at other levels, such as community and society, have been generally ignored [40]. In Morocco, as in other conservative Arab countries, studies on contextual factors affecting adolescent risky sexual behavior leading to STIs are scarce [8, 19, 40]; This is mainly due to the cultural, social, and even religious barriers in these countries, which make conducting studies on sexual health of adolescents a difficult task. The present paper aimed to fill this gap in knowledge by exploring factors that influence adolescents’ sexual behaviors in a conservative country using a socio-ecological perspective. We conducted a qualitative study to explore perceived risks and protective factors of risky sexual behaviors leading to STIs in adolescents and to identify opinions on ways to improve the sexual health of Moroccan adolescents.

## Methods

A qualitative study using focus group discussions was designed to provide a deep understanding of the participants views and experiences on the subject. We collected data via focus group discussions (FGDs) with three different categories of participants (adolescents, parents, and teachers). This design, which allows exploring the views of different groups in similar or different categories [44], suits our purpose and enables us to consider three different views of three categories of participants [45].

### Participants and study procedures

Our sampling process was based on the guidelines of the project sample size for thematic analysis recommended by Braun and Clarke [46], which categorize suggestions by the type of data collection and the size of the project (‘small’, ‘medium’, or ‘large’). Given that this study was part of a larger project, we followed the recommendation of recruiting 3 to 6 focus groups (with 4 to 8 participants per group) for each category of participants, totaling in 12 to 48 participants per category. We conducted 8 FGDs with 56 adolescents (28 boys and 28 girls), randomly recruited from two public middle schools (disadvantaged and advantaged, according to socio-economic level) in Taza city, Morocco, from May to July 2016. From each school, adolescents, in their last year of middle school aged 14 to 16 years, were enrolled. This age group was selected because their school curriculum includes topics related to health risk behaviors. In addition, we conducted 5 FGDs of parents (5 females and 21 males) who were selected based on their voluntary participation. Teachers of disciplines related to health risk behaviors (i.e. Life and earth Science, Familial education, Islamic education, Arabic language, Physical education) from the selected schools were randomly invited to participate in the study. They were 13 males and 5 females grouped into 4 FGDs. Further participants characteristics are presented in Table 1. To respect the conservative norms of Moroccan society, we used single-sex focus groups. Sampling continued until data saturation was achieved, which is defined as the point where data collection and analysis cease to provide new information and the same themes are recurring, so that little or no change is given by additional data during two consecutive focus groups for each category of participants.

**Table 1:** Focus Groups participant characteristics

Focus group characteristics	Adolescent group (*n* = 56)	Parents group (*n* = 26)	Teachers group (*n* = 18)
# of Focus groups	8	5	4
# of Participants per focus group	7	5 or 6	4 or 5
# of Participants (%)			
Male	28 (50)	21 (80.8)	13 (72.2)
Female	28 (50)	5 (19.2)	5 (27.8)
Age range, y	14–16	30–60	30–60
Education/Parents			
Fathers (% Illiterate)	4.0	NA^a^	NA^a^
Mothers (% Illiterate)	36.4		

^a^*NA* Non-applicable; data not collected

Each group discussion was conducted by two researchers at locations within the schools while observing adequate confidentiality. One of the two researchers moderated the discussions while the other recorded the conversation, took notes and observed group interactions and nonverbal individual behaviors. At the beginning of each session; the group moderator briefed participants about the study objectives and the nature and procedure of the discussions while reassuring confidentiality and participants rights. At the end of the sessions, all key points raised were concluded and participants were invited to add or clarify their ideas. Each group discussion lasted about 45 to 60 min.

### Data collection instruments

The FGDs were semi-structured and were developed by the research team based on the socio-ecological model as a theoretical framework [27]. There were three different guides of open-ended questions (adapted to each group of participants) used to explore perceived risks and protective factors of risky sexual behaviors leading to STIs in adolescents at the individual, relationship, community, and societal levels as well as their intervention preferences (Table 2). The questions designed for adolescents and parents addressed their perceptions regarding barriers and facilitators for risky sexual behaviors and STIs in adolescents and their intervention preferences. The FGD guide for teachers was designed to explore their perceptions regarding barriers and facilitators of risky sexual behaviors with a focus on the context of school and education curricula. Suggestions on interventions were also explored alongside teachers. Interview questions were tested in small groups of each category of participants to identify wording issues. In addition, a brief questionnaire was used to collect participants’ socio-demographic characteristics (gender and age).

**Table 2:** Sample of open-ended questions in focus group discussion guides

Adolescents	Parents	Teachers
STI-related risk and protective factors		
1. What are the sexual-related activities among teens in your community (especially risky activity)? 2. How are these sexual-related problems (STIs) affecting young teens in your community? 3. In your expert opinion, what risk (individual, family, peer, community, society) causes teens to start risky sexual activity? 4. In your expert opinion, what protective factors (individual, family, peer, community, society) prevent teens from starting risky sexual activity?	1. What are the sexual-related activities among teens in your community (especially risky activity)? 2. How are these sexual-related problems (STIs) affecting young teens in your community? 3. In your expert opinion, what risk (individual, family, peers, community, society) causes teens to start risky sexual activity? 4. In your expert opinion, what protective factors (individual, family, peer, community, society) prevent teens from starting risky sexual activity?	1. What are the sexual-related activities among teens in your community (especially risky activity)? 2. How are these sexual-related problems (STIs) affecting young teens in your community? 3. In your expert opinion, what risk (individual, family, peers, community, society levels) causes teens to start risky sexual activity? 4. In your expert opinion, what protective factors (individual, family, peer, community, society levels) prevent teens from starting risky sexual activity? 5. What is the role of the school in either increasing or decreasing risky sexual activity?
Preference of effective intervention models		
1. What do you think would work within the levels (individual, family, peer, community, society) to prevent teens from getting STIs?	1. What do you think would work within the levels (individual, family, peer, community, society) to prevent teens from getting STIs?	1. What do you think would work within the levels (individual, family, peer, community, society) to prevent teens from getting STIs?

### Ethics approval and consent to participate

We received ethical approval from the Faculty of Medicine and Pharmacy of Casablanca Research Ethics Committee and the National Control Commission for the Protection of Personal Data (A-RS-193-2015). After explaining the objectives of the study, written informed consent was obtained from all participants before study enrollment. Concerning adolescents, written informed consent was obtained from their parents or their legal guardians in addition to a verbal consent from each adolescent. Confidentiality of study participants was ensured at all procedures. We have guaranteed their rights to freely retain information if they were embarrassed to give it. Only the lead investigators had access to the complete tapes. The names of participants were kept separately from tape recordings, transcripts, and field notes, and secured where only the principal investigator had access.

### Data analyses

All discussions were audio-recorded, transcribed verbatim, and translated into English by the authors. The research team verified the accuracy of all transcripts by listening to the recordings while reading transcripts. The three separate datasets of adolescent, parent, and teacher discussions underwent thematic analysis [47], thus providing technical reconnaissance and a rich and comprehensive exploration of recurring themes within the data. Inductive thematic analyses allowed us to explore participants views without trying to fit these into a preexisting coding frame [47]. We also used a semantic level of thematic analysis to analyze our data [47]. In other words, most of the statements in the focus group were taken at face value and were not interpreted for an underlying meaning in the development of our themes [47].

Our dataset underwent the six phases of thematic analysis as recommended by Braun and Clarke [47]: familiarization with data, generation of initial codes, searching for themes, reviewing themes, defining and naming themes, and producing the report. Familiarization with data was initiated during the data collection phase, as we personally conducted the transcription. In addition, we were intensely involved in the data by listening to audio recordings and reading transcripts several times. During the second phase, common ideas were identified and interesting patterns were noted. This phase involves identifying codes from the data. The coding process was iterative; to ensure transparency and reliability, all transcripts were coded systematically by two researchers independently. The research team discussed in detail the initial coding and interpretation of the transcripts. All coding differences were resolved and the codes were refined and grouped into key themes that capture something important about the data in relation to the research question. The next phase consisted of reviewing themes, which was an iterative process, as we constantly moved back and forth between the selected extracts from the data and the entire dataset to ensure the applicability of themes. Once the themes had been established, they were defined and labeled in such a way to be conceptually distinguishable from each other. Next starts the process of writing the final report by making a set of fully-developed themes with vivid examples reflecting the essence of each theme and contributing significantly to answering the research question. Finally, the overreaching themes were categorized according to the socio-ecological model [43], allowing us to explore any perceived risks and protective factors that influenced adolescent engagement in risky sexual behaviors leading to STI acquisition.

## Results

All participants stated that many adolescents engage in risky sexual practices. A number of factors emerged that seemed to positively or negatively influence these behaviors. These factors can be discussed under five different themes:

### Theme 1: risky sexual practices and STIs (individual level)

Most of the adolescents perceived that they lacked information regarding sexuality and STIs, including HIV, particularly about STIs mode of transmission and prevention methods. They also revealed their need to know more about sexuality and STIs. This opinion was shared by most parents and teachers within the discussion groups.

*“The majority don’t have much knowledge about this topic. They don’t know that AIDS can be transmitted sexually or through other means such as (a razor blade, blood…); we must know more about this…” _ Boy*

*“Neither my sons nor their siblings know about this topic.” _ Mother*

*“They are not aware of the dangers of the sexually transmitted diseases and don’t know how to protect themselves.” _ Male teacher*

Some parents stated that they also had inadequate knowledge regarding STIs, including the type of infection, their mode of transmission, and preventive methods. One father stated, *“Personally speaking, I don’t know any of the sexually transmitted diseases.”*

Because of the great emphasis on HIV/AIDS by public health officials via extensive and global awareness campaigns while neglecting the other STIs; many of the adolescents’ participants knew only about HIV/AIDS and ignored other STIs. They also stated that these infections are transmitted through illegal sexual activity and not by other ways such as unprotected sex, blood transfusion, sharing needles, and drug injection. This misconception is adopted by the conservative Muslim community, which prohibits premarital sex and consider it a sin “punishable by serious diseases”. One boy stated, *“If a person does a sexually illegal relation with someone who has AIDS, he would be infected and would never recover. AIDS has no cure.”*

Few adolescents understood that the use of condoms could protect against STIs and pregnancy. In addition, some teachers perceived that the lack of money to buy condoms increased the risk of STI acquisition among adolescents. A male teacher stated, *“…most of teenagers make sex without condom because they can’t afford it.”*

Knowledge regarding sexuality and STIs was cited to be gained through several sources, including media, peers, internet, awareness campaigns, and family. The most cited source was school, especially biology classes, as reported by many adolescents and parents. One boy said, *“I know about this topic from school. We study about STIs and how to protect ourselves from these diseases in biology.”*

Furthermore, most participants in all three groups indicated that carelessness, curiosity, and the adolescent tendency to focus on the immediate, rather than the long-term, consequences of their behavior make them vulnerable to experiment, including with risky sexual activity. The pursuit of sexual pleasure was also considered as a facilitator that could lead to engagement in risky sexual activity despite being aware of the health consequences of these behaviors.

*“This happens because of teenagerhood. In this period, the teenager tries to prove themselves. Some teenagers try drugs; others get involved in sexual relations with the opposite sex.” _ Boy*

*“There are people who are aware of this topic but can’t control their desires.” _ Girl*

*“There are people who don’t give this topic much importance and care only about instantaneous pleasure.” _ Mother*

*“Adolescents are vulnerable to these diseases because they ignore the consequences of illegal sex. They are also blind by the desire to discover something they have never done before.” _ Male teacher*

Other adolescents and teachers agreed that emotional relationships between boys and girls increase the chance that adolescents participate in such risks and would lead to increased acquisition of STIs and HIV. They also reported that some girls with low socio-economic status engage in transactional sex in exchange for money, especially with older men.

*“There are girls who offer their body because they need money to buy clothes and brag to their friends. They can even make sex with men who are very old.” _ Girl*

*“Some girls are convinced of the financial returns of prostitution, so they are reluctant to get married.” _ Female teacher*

The increased use of illicit drugs and alcohol during adolescence was viewed by many teachers as a facilitator to adolescent involvement in risky sexual behaviors and increased risk of contracting STIs when under the influence of these substances. A female teacher stated, *“Addiction to drugs is one of the causes of these sexual behaviors.”*

In contrast, some adolescents stated that their awareness of the dangers of risky sexual activity particularly STIs, pregnancy and shame kept them abstinent before marriage. As one girl stated, *“People who don’t make sex are aware of its dangers. They know that they might get pregnant or get infected with AIDs and they will be marginalized by society.”*

Other parents believed that an adolescent’s fear of diseases, especially AIDS, and the desire to keep healthy can be protective factors for adolescents to avoid risky sexual activity and decrease STI acquisition. As one father highlighted, *“Fear from the diseases that destroy many aspects of the human being.”*

### Theme 2: the adolescent’s social domain (relationship level)

This theme reflects participants perceptions about the influences of social relationships that could increase or decrease the likelihood of an adolescent to engage in risky sexual activity. This main theme included two subthemes: family influences and peer influences.

#### Family influences (parental involvement)

In all FGDs, most participants stated that the lack of sexual health discourse between parents and their sons and daughters could lead to increased adolescent engagement in risky sexual activity. Participants stated that the reason for the lack of communication about sexuality and STIs was because these were considered taboo subjects in a conservative society. Having such a conversation was seen as a lack of respect and perhaps an encouragement for these practices. Some parents and teachers acknowledged that the lack of awareness of parents regarding these problems makes this discourse difficult.

*“I don’t dare to discuss this topic with my parents; it’s a taboo.” _ Boy*

*“Families don’t talk about this issue because it is a taboo. Some parents don’t talk about this issue because they know nothing about it.” _ Father*

*“There is a communication gap in the family. The parents don’t sensitize their children about this topic.” _ Female teacher*

Some adolescents and parents believed that social problems such as divorce, poverty, and parental neglect, may push adolescents to engage in risky sexual behaviors as a way to escape their status quo.

*“Domestic problems and the nonchalance of parents make adolescents turn to illegal sexual relations.” _ Mother*

*“Teens turn to drugs when their parents are divorced and don’t give them the time they deserve.” _ Girl*

Some teachers believed that parental monitoring and guidance were lacking, including parents trusting their teens too much, leaving them unsupervised, and giving them excess idle time, thus diverted their attention to risky behaviors. A female teacher emphasized, *“Lack of supervision and care by parents and guardians as well as leaving free time for the teenager pays for these behaviors.”*

In contrast, many adolescents perceived that parents who play a positive role in providing information and counseling regarding sexuality and STIs, especially mothers, kept adolescents away from risky behaviors. One girl stated, *“Education and listening to parental advice is important. My mother advises me every day.”*

#### Peer influences

Peer influences were reported by many adolescents and teachers as both encouraging and hindering risky sexual behaviors among adolescents. Adolescents in peer groups whose members engage in risky behaviors often adopt a similar behavior.

*“Some teens are easily influenced by the risky sexual behavior of their friends and even imitate them to gain their favor.” _ Female teacher*

*“The bad friends, the ideas of some friends, they just tell you to try and you'll see that it's fun and exciting or something like that.” _ Boy*

*“Students don’t choose good friends. Their choice of friends influences their lives. For instance, bad friends can encourage the person to do illegal sex.” _ Male teacher*

In contrast, many adolescents emphasized that having friends who do not have early sexual intercourse can positively influences adolescent sexual activity by protecting them from engaging in such behaviors.

*“Having good friends who don’t engage in risky sexual behaviors is also important in this regard.” _ Girl*

*“Many students do not engage in such behaviors because they have friends with healthy behaviors; for example, most of my friends do not have sexual relations. So, I don’t engage in risky sexual activities.” _ Boy*

### Theme 3: the role of school (community level)

School was viewed as a salient contextual factor that can negatively or positively influence adolescent risky behaviors. Participants in all three FGD categories agreed that schools were playing a limited role in raising adolescent awareness about sexuality and STIs and changing their risky behaviors. Participants stated that this was mainly because information and awareness provided at schools are inadequate compared with temptations offered to adolescents on the Internet, the media, and in society at large, by providing misleading information that may stimulate their desire to engage in risky sexual behaviors.

*“…we study these sexual behaviors but we don’t give what we study a lot of importance unless the person who teaches it is convincing.” _ Girl*

*“The provided information and advice are insufficient compared to the temptations offered on the Internet and the means of communication that trigger the instincts.” _ Father*

*“The school is trying to reduce these sexual behaviors, but this role remains very limited.” _ Female teacher*

Most teachers and parents believed that the sexual health content provided by schools for adolescents is insufficient, especially in terms of learning objectives and allocated time. In addition, participants perceived that school sex education classes could provide opportunities for interaction with positive models and participation in activities that could increase self-esteem and feelings of empowerment, thus stimulating youth to make healthy sexual decisions.

*“There is a lack of programs that have clear objectives and address these phenomena by raising awareness of it. The existing school programs are limited to the biological and physiological side of the topic and use traditional methods despite the technological evolution.” _ Female teacher*

*“There is no school subject that teaches sexual culture at any educational level. Sexuality is part of life; we should study it especially that there are diseases that can be transmitted sexually.” _ Male teacher*

*“Textbooks don’t tackle this issue as it should be done.” _ Mother*

Teachers felt that communication about sexuality and STIs with students was scarce because it is still considered a taboo, and such discussion is not always easy because students feel embarrassed and unable to communicate or interact positively with their teachers. As one male teacher specified, *“Even when you talk about this topic and want to sensitize them, they don’t interact because this issue is a taboo.”*

In contrast, many adolescents and teachers believed that the classes of “Islamic education” did raise awareness about sexuality and protected adolescents from engaging in risky sexual behaviors by reinforcing religious beliefs.

*“Islamic education tackles this topic, but only from an Islamic perspective.” _ Girl*

*“I tell them that we learn Islamic education not for the sake of knowledge but for the sake of applying it in our daily life with our wives.” _ Male teacher*

Furthermore, extracurricular activities (especially school clubs, sports, drawing, and theater) were another important protective factor within the school system that emerged from parent and teacher discussions, whereas adolescent groups did not mention this protective effect of extracurricular activities.

*“To sensitize adolescents about these behaviors through their participation in school club activities, such as the publication of wall magazines and awareness programs on these subjects.” _ Father*

*“Many students are consciously away from these behaviors due to their participation in several activities of health and sports clubs as well as theater and drawing, which are organized at the school as extracurricular activities.” _ Male teacher*

### Theme 4: media, including internet and social media (societal level)

In all three groups, most participants agreed on the negative role of television, movies, advertisements, the Internet, and other social media in increasing adolescent sexual risky behaviors by showing teenage characters with multiple emotional and sexual relationships. In addition, some students and teachers agreed that new technologies, such as smartphones, make it easier for teenagers to engage in risky sexual behavior by providing a wide range of pornographic content and facilitating communication and “sexting” between boys and girls at an early age via social media. Facebook and WhatsApp were both mentioned as negative facilitators.

*“Teenagers are affected by pornographic films and advertisements in television and in media.” _ Boy*

*“When you open a website to look for something, ads pop up displaying sexual content. Someone who doesn’t control themselves will click for pornography.” _ Girl*

*“The availability of technological means facilitates the access to everything that is pornographic or inadequate for adolescents; there is also the exchange of pictures and pornographic videos on social networking sites such as Facebook and WhatsApp… which increase risky sexual behaviors.” _ Male teacher*

*“Providing easy technology that provides access to pornographic sites.” _ Father*

According to some students and parents, the role of the government in raising awareness and protecting young people from these risky behaviors through media is inadequate and not serious. While media outlets were perceived to contribute negatively to the spread of these risky sexual behaviors within the community, especially among adolescents who are most vulnerable, a boy said, *“The government is not playing its role in sensitizing people through social media, and television doesn’t display programs that sensitize either.”*

### Theme 5: socio-cultural norms (societal level)

Participants agreed that changes in social norms within the conservative Moroccan society have negatively influenced adolescent sexual behaviors. Participants also believed that many risky sexual behaviors have spread and have become acceptable by teenagers despite their contradiction with Moroccan norms and religious instructions.

In all three FGDs, most participants consistently believed that the normalization of risky behaviors have occurred within the Moroccan society, including normalization of risky sexual behaviors. Teenagers who do not conform to these new norms are now considered abnormal and fossils. Participants believed that teenagers are unsafely exposed on a daily basis to an array of tempting practices, including revealing dress habits, which encourage unthoughtful engagement in dangerous sexual behaviors. Similarly, they stated that there are more romantic relationships between boys and girls and increased ease in dating, even at an early age, influencing and facilitating the engagement of adolescents in such risky behaviors, hence, raising STI and HIV acquisition.

*“There are a lot of adolescents who are making sex as if they are married.” _ Boy*

*“The status quo and the sexy clothes that girls wear and sexual harassment influence either.” _ Father*

*“They behave in that way because they feel that society accepts their sexual behaviors. The spread of some misconceptions among adolescents, the freedom to use one’s body, and the lack of respect for community norms and traditions such as chastity and compassion are the causes of these problems. The practices that were once for us shameful and unacceptable are now seen as modernity and progress.” _ Male teacher*

One female teacher indicated that harassment of girls on a daily basis at schools and in the streets in general makes them vulnerable to these risky behaviors under the influence of constant pressure from the harassers. *“Under pressure and sexual harassments in the streets and at school, girls might get involved in illegal sexual relations.”*

Most respondents in all three categories stated that weakened religious and moral beliefs among adolescents can lead them to engage in risky sexual behaviors, as they lacked the ethical considerations that enable them to distinguish healthy from unhealthy behaviors.

*“In some cases, ignorance of religious instructions and the absence of morality drive them to such risky behaviors.” _ Girl*

*“In my opinion, adolescents are not sufficiently initiated to religion by claiming that they are still young, which contributes to their commitment to these sexual behaviors.” _Female teacher*

*“The lack of awareness of religious and moral education among adolescents leads to these dangerous behaviors.” _ Father*

Conversely, many respondents in all three groups said that adolescents with strong religious and moral beliefs were safe from drifting toward behaviors that could increase the risk of STIs.

*“Religious people will stay away from this; however, the ones who are ignorant won’t.” _ Boy*

*“Adolescents who know the tenets of their religion regarding these behaviors don’t engage in such risky practice.” _ Mother*

*“Also, I think that a teenager who receives appropriate religious education within his/her family will not be influenced by such risky behaviors because they contradict the teachings of his/her religion.” _ Male teacher*

### Perceptions regarding effective interventions for reducing adolescent risks for STIs, including HIV

Participants suggested a number of possible interventions to improve sexual health and reduce HIV/STI risk among adolescents. These suggestions can be grouped according to the socio-ecological levels. Table 3 presents illustrative quotes from different focus groups in support of intervention preferences.

**Table 3:** Perceptions of effective intervention preference for reducing adolescents’ risks for STIs including HIV according to socio-ecological model levels – individual, relationship, community and societal

Socio-ecological model levels	Adolescent group	Parents group	Teachers group
Individual level	*B1: “Education at an early age must be based on values, ethics, and religion, away from the forbidden things of all kinds, and we must also use these values to implement successful interventions.”* *G1: “Teaching social skills to the teenager in order to be able to manage and control his desires.”*	*F1: “We need programs that educate young people and spread a culture of chastity and modesty.”*	*M.T1: “I believe that strengthening religious beliefs in adolescents can give them immunity and protection against dangerous behavior.”*
Relationship level	*G2: “Parents should supervise their children and know what kind of friends they have, and what they watch on TV and on the Internet.”* *G3: “… Prosocial students should be involved in such interventions as they are a good example and can positively influence their colleagues.”*	*F2: “Parents should be made aware about all these issues related to sex education and should learn the ways and skills required to communicate with their children in these subjects, which is still considered taboo.”*	*F.T1: “We must involve parents of students and organize meetings with them so that the behaviors observed in their children can be followed and thinking together about ways of dealing with these behaviors.”* *M.T2: “Parents should know more about STI transmission and prevention methods so that they can educate their children about these behaviors that are harmful to their health.”*
Community level	*B2: “I think sex education should be included in the curriculum through a school subject that specializes in all risky behaviors that can affect teenagers.”* *G4: “They should add school subject to the curriculum about sex education and unhealthy behaviors and how to avoid them. They should also organize programs to educate adolescents because they have a lack of knowledge regarding STIs and they are more likely to engage in adultery.”*	*F3: “School officials should encourage students to practice sports by organizing sports competitions so that they can fill students’ spare time with useful activities and thus avoid dangerous sexual behaviors.”* *M1: “Outreach programs must be implemented in partnership with school clubs and associations. In addition, the involvement of students themselves in such intervention by making presentations on STIs and methods of prevention so that they develop a sense of responsibility and can avoid risky behavior.”*	*M.T3: “Sex education must be included in school curricula of all levels and specialties, whether literary or scientific ... we can also devote more time to these topics and present them in an interactive way by involving students in order to become more aware of these behaviors and to avoid them.”* *F.T2: “Modern technological means must be used in sex education because we notice when we carry out activities related to sexually transmitted diseases and we use images and videos, we can see that students are more interested and more influenced.”* *M.T4: “There are, of course, health clubs that have to organize various activities on AIDS in particular and other sexually transmitted diseases with the help and partnership of the Ministry of Health.”*
Societal level	*G5: “Promote awareness among adolescents by increasing health programs in the media, which must be provided with the help of specialists and celebrities who have an impact on adolescents.”* *G6: “The government must block these pornographic and ugly sites, and it must also fight adultery in order to limit the spread of these diseases.”*	*F4: “Even Moroccan television has to offer respectable Moroccan films, not Turkish translated films or Indian films that are offered to Moroccans, which are not their culture.”* *M2: “Media should provide targeted programs that show adolescents the risks of sexually transmitted diseases.”* *M3: “I think this is everyone’s responsibility and that the state should also impose sanctions on any minor who has premarital sex.”* *F5: “The Ministry of Health must conduct confidential medical screening on students.”*	*F.T3: “In the media, there should be special programs to raise awareness of the seriousness of these sexually transmitted diseases, not only at the social level but also at the economic level.”* *M.T5: “In this area, the Ministry of Health has to move and organize many awareness programs continuously, and the state must provide cultural centers for young people to build their personalities and to fill their leisure time.”*

*G* Girl, *B* Boy, *F* Father, *M* Mother, *M.T* Male teacher, *F.T* Female teacher

#### Individual level

In all three FGDs, many participants suggested that implementing interventions aiming to enhance religious beliefs, knowledge of sexuality and STIs, and safer sex skills could have positive outcomes on adolescent awareness and behaviors. Thereby, such interventions could promote safe sexual practices and reduce risky ones.

#### Relationship level

Most participants in all three groups emphasized that parental involvement in interventions would have a positive effect. These interventions would educate parents themselves with the correct information about STIs risks and teach specific parenting skills and dialogue techniques so that parents could communicate actively with their children on this topic. In addition, they insisted that parents must play a major role in monitoring and supervising their children, by overseeing their access to the Internet and the related mobile devices. They also stressed that parental guidance is necessary and parents must ensure that their children watch age-appropriate television and YouTube materiel without any pornographic content. Parents should also have a say in making friendship choices of their children.

Some students stated that having ideal and socially active peers in interventions would have a positive effect in enhancing responsible sexual choices because teens communicate well with each other without formalities and can influence each other positively.

#### Community level

Most participants in all three FGDs stressed that schools could play a central role in the success of interventions about sexuality and STIs. First, schools should intensify sex education in terms of additional allocated hours and through development of adolescent communication skills related to sexuality. In addition, it was suggested that modern technologies should be used to facilitate the transmission of this information. Other adolescents and teachers suggested that sexual education should be a basic school subject in the curriculum with enough hours and clear objectives and offered by well-trained teachers, especially for adolescents who are vulnerable to these risky behaviors.

Second, it was suggested by many teachers and parents that schools should use extracurricular activities to raise awareness and improve student social skills. The involvement of adolescents in cultural, environmental, and sport clubs offers opportunities for the youth to develop abilities and skills that help them to avert harmful behaviors, give them a sense of responsibility, and increase time for positive activities.

Other participants in all three categories stressed that awareness campaigns can be organized in partnership with school clubs and civil society associations by making students an effective contributor to these campaigns, with the inclusion of physicians and specialists to mark a greater impact on students. Similarly, some parents suggested organized field visits to hospitals so that students closely witness cases of STIs and talk to healthcare providers to depict real and vivid examples about the phenomenon.

#### Societal level

Participants from all three groups indicated the effectual role that could be played by the media in reducing STIs risks. They agreed that media companies should limit movies and series that encourage risky sexual behaviors and that media groups must take some responsibility in raising awareness among adolescents through various programs presented by physicians, psychologists, and celebrities who may have positive influences on teenagers.

Students and parents agreed that the government should combat misconduct and sex outside of marriage norms by imposing severe penalties on those who violate them. They also stressed that the government must block pornographic sites that are easily accessible by adolescents.

Other parents and teachers suggested that the Ministry of Health should help raise adolescent awareness about risky sexual behaviors and STIs by organizing targeted and ongoing sensitization campaigns at school. They also suggested that the Ministry of Health could conduct confidential screening campaigns to detect STIs in students. Furthermore, some parents stressed the need to fill adolescent free time and provide them with recreational outlets such as youth centers and theaters and promoting sport clubs so that adolescents could release energy, develop their communication and social skills, and gain self-esteem and sense of responsibility.

## Discussion

To date, this is one of few studies conducted in Morocco that provides an initial qualitative understanding of risks and protective factors affecting risky sexual behavior and STI acquisition among adolescents based on the perceptions of adolescents, parents, and teachers. These findings are supported by previous studies on ecological theories of human development, showing that determinants of sexual behavior belong to different levels [32, 48]. The present study also unveiled a number of suggestions about preferred interventions to reduce these risks in adolescents, which can be classified and applied at multiple levels from the individual to the societal.

### Individual level

In agreement with previous studies [21, 25, 34, 39, 40], incorrect or a lack of knowledge about sexuality, modes of STI transmission, and STI protection were viewed by most participants in all three categories as a major facilitator of adolescents to engage in risky sexual behaviors by making them vulnerable to unsafe and inappropriate reproductive health choices, which can have detrimental effects on their reproductive health. This lack of awareness was due to limited sex education, especially within the family, as many parents revealed that their knowledge and information regarding STIs were insufficient. Although the school was cited as the main source of information for adolescents by almost all participants in this study, schools remained insufficient as they tended to present information in a traditional way and focused more on biological content. Furthermore, although some adolescents had sufficient information on prevention methods against STIs, they struggled to protect themselves as they could not afford contraceptive methods like condoms, as reported by many teachers. Financial barriers limiting access to reproductive health services among adolescents have been widely documented [9]. On the other hand, similar to other reports on the protective effects of adolescent awareness on STIs [21, 23, 49], some adolescents in our study believed that awareness about the dangers of risky sexual activity, including STIs, pregnancy, and shame, can be barriers to adolescent engagement in early and risky sexual activities.

As mentioned previously by several studies [19, 20, 31, 49–51], adolescents undergo physiologic, psychological, and emotional changes that are characterized by curiosity, carelessness, and a high level of sexual pleasure-seeking, which we found to be among the main causes for adolescent to engage in risky sexual behaviors. This can be explained by the increase in risk-taking during adolescence as a result of changes in the brain’s socio-emotional system leading to increased sensation seeking [51]; these changes can contribute to the tendency in adolescents to focus on immediate desires without considering the long-term consequences of their behavior on health. This was heightened by the many romantic relationships between boys and girls in this age group, as highlighted by several adolescents and teachers. Likewise, as reported previously [20, 24, 28, 34, 41, 42], many of our teachers agreed that drug and alcohol use, which is common during this period, can lead to adolescent involvement in these behaviors by influencing their positive choices and decision-making regarding sexual behaviors. It was also stated that poverty and lack of financial resources of some girls, can lead them to exchange sex for money to afford their needs, which may increase the risk of STI acquisition among these girls. These findings are consistent with other studies among adolescents, in which girls were at high risk of STI acquisition [20, 21, 24, 34, 41]. This issue requires interventions at the societal level that reduce socio-economic inequities, impose policies and laws that protect women’s rights, and punish anyone who exploits the financial needs of girls to satisfy their sexual desires.

In contrast, some parents reported that fear of STIs and desire to maintain good health played positive roles in adolescents, who then abstain from risky sexual behaviors before marriage. This was in line with previous studies, which showed the protective role of awareness about the consequences of unsafe sex and the fear of STIs [21, 24, 49]. Thus, increasing the awareness of adolescents about sexuality and STI consequences and building their decision-making skills with regard to risky behaviors can have positive effects on adolescent sexual health.

### Relationships level

Within an adolescent’s social environment during growth and development, family and peers are important sources of protective and risk factors for risky sexual behaviors and STI acquisition. With regard to family influence, as reported elsewhere [11, 27, 31, 50, 52–54], the lack of discourse on sexual health between parents and their adolescent children and inappropriate parental knowledge about these issues were shown to be important facilitators for risky sexual practices in adolescents. Parents also stated feeling embarrassed and shy to discuss these taboo issues with their children while some of them believed that such discussion would encourage sexual activity. In accordance with previous studies [11, 27, 54], such barriers perceived by parents and their low self-efficacy in communicating about HIV and STIs with their children, may reduce their involvement in sex education and increase the likelihood of STI acquisition in adolescents. Similar to other studies [52, 55], we found that parents also tended to underestimate the sexual risk behaviors of their own children, further minimizing necessary communication. In addition, this study corroborated findings from previous studies conducted among adolescents [20, 56, 57] in which social problems, including divorce, poverty, and neglect, were viewed as detrimental situations that affected parent-child relationships, created stressful conditions, and weakened adolescent personality, making them vulnerable to risky behaviors. Moreover, as reported elsewhere [50], some teachers perceived that the lack of parental monitoring contributed to adolescent engagement in risky sexual behaviors. Indeed, exposure to an authoritative parenting style that also included high levels of warmth, involvement, communication, and monitoring decreased the likelihood of adolescent predisposition to risky sexual behaviors [58]. This can be also explained by the fact that parental monitoring could reduce negative influences of peers, as friendship choices are influenced by authoritative parenting practices, and possible unsupervised situations and idle time where risky sexual activity can occur are reduced [34, 58]. In contrast, many adolescents viewed family as a protection system, especially for those who received good counseling within the families and communicated well with their parents, especially with their mothers. This gendered sex communication in which parent and child gender are important factors, especially concerning mothers, has been supported in the literature; mothers tend to be more proactive in their discussions about sexuality, they cover more topics, and they are easy going when they talk about sexuality compared with fathers [11, 27, 54]. Adolescents within this protective environment could discuss sexual problems with their families in stress-free and outgoing emotionality; hence, influenced by their parent’s values and norms, in meeting their expectations to avoid these behaviors, as documented previously [39, 59].

Many adolescents and teachers pointed out that peer influences can also increase or decrease risky sexual behavior among adolescents. Since peer acceptance is widely sought and teens tend to believe that what their peers do, is what defines the normal behavior of adolescents, some can adopt the negative norms of their peer networks, including risky sexual behaviors. The role of peers in influencing an adolescent’s choice to engage in unsafe sex has been well established [31, 33, 34, 40, 42]. This influence is especially important during adolescence, where teens set the process of distancing themselves from their parents and prioritize the values and behaviors of peers over those of their own families, especially in cases of poor parental relationship. In addition, risk-taking increases as a result of changes in the brain’s socio-emotional system during adolescence, particularly in the presence of peers [51]. However, and similar to other studies [39, 49], adolescents who have close and supportive friends who do not engage in early sexual practices are less likely to engage in such behavior. The protective effects of family and peers can be exploited outside the family and social context, particularly at school, by ensuring a supportive and positive school environment that can help prevent or reduce risky sexual activity.

### Community level

Within the community level, the role of school emerged as an important contextual factor that can negatively or positively influence adolescent risky behaviors. Similar to that previously reported [11, 27, 31, 60], most participants in all three groups agreed that the current school’s role in raising awareness and changing practices regarding sex and STIs was inadequate and not effectual enough to offset temptations offered to adolescents in the society. Most teachers and parents stated that the sexual health content in the school curriculum was insufficient and lacked clear objectives and enough allocated hours. Additionally, in alignment with previous research [61], we found that the curriculum focused more on traditional academic material rather than the risky behaviors of adolescents. Teachers also added that communication about sexuality and STIs with students was rare, as they felt embarrassed when talking about this topic, which is considered a taboo in Moroccan society; such cultural factors seemed to contradict the whole purpose of sex education, as reported elsewhere [11, 19, 27].

Other participants reported that Islamic education school subject appears to play a protective role in the sexual health and behavior of adolescents. Indeed, several studies revealed that strengthening religious beliefs and spiritual activities resulted in adolescents making positive decisions regarding risky behaviors [56, 60, 62]. Moreover, extracurricular activities (especially school clubs, sports, drawing, and theater) were important protective factors that emerged from discussions with parents and teachers, supporting previous studies that highlighted the importance of engaging in school club activities [60]. The feeling of belonging is important to development in adolescents, encouraging responsibility for their own actions and their team and creating a sense of attachment within the school system, which can be used to protect teens from risky sexual practices [60]. Adolescents may not mention extracurricular activities as a protective factor because they view this as entertainment and do not consider these activities a health benefit; however, parents and teachers do recognize the importance of these activities in the development of adolescent personality and health. As previously reported, parents preferred to encourage their children to participate in extracurricular activities and to make sure that they have well-behaved friends instead of directly educating them about the dangers of risky sexual behaviors [63].

These finding about the school system emphasize the need to introduce sex education as a school subject in the curriculum. Such opportunities could provide interactions with positive role models, increase self-esteem and the sense of empowerment, and encourage young people to make healthy sexual decisions.

### Societal level

Within the societal level, participants reported on the role that media outlets and changes in social norms play in adolescent sexual behaviors. Similar to previous studies [24, 28, 56, 62, 64], participants in all three FGDs stated that media outlets promote risky sexual behavior by displaying sexual content as normal behavior, especially in television series and movies. In general, the media tend to describe sex as safe entertainment, without showing the risks and consequences of risky sexual behavior. In addition, adolescents who watch and hear a lot about sex in the media are more likely to have early sex than those who are rarely exposed to sexual content [24, 28, 64]. Consistent with previous studies, adolescents and parents in the current study believed that the media did not adequately raise awareness about risky behavior among adolescents [56, 62]. This lack of seriousness of messages from the media is especially detrimental to conservative societies where sexual topics remain taboo; within conservative societies, the media can be an important source of sexual information for teens. Furthermore, as reported elsewhere [42, 56, 62, 65, 66], some students and teachers agreed that adolescents spend more time than ever before on new communication technologies; therefore, negative messages from social media can influence adolescent behavior and increase risky sexual behavior. For example, several investigations showed that social media applications can provide access to a wide range of pornographic images and videos; these devices can also facilitate communication and sexting between boys and girls at an early age, which may cause early interest in sex and accelerate the normal rise in sensation seeking during adolescence [42, 64–67].

Furthermore, as reported in other contexts, due to globalization, access to technology, and international media, remarkable changes in social norms have resulted. These changes have particularly affected conservative societies, such as Morocco, and have negatively influenced the sexual behaviors of adolescents [10, 19, 56, 64]. Participants in all three groups stated that risky behaviors, including sexual behavior, have become accepted as normal within the society. This widespread normalization of sexual activities seems to have allowed acceptability of premarital sex for adolescents in Morocco despite being contradicting to their traditional cultural and religious roots, as documented elsewhere [10, 19, 28, 56]. In addition, some teachers indicated that harassment of girls on a daily basis can lead them to engage in risky sexual practices. This finding highlights gender-related power differences that expose adolescent girls to the risk of contracting STIs. Such societal power relations tend to disadvantage girls, by making them vulnerable to transactional and coerced sex [10, 41].

Similar to other empirical studies [24, 56, 57, 62], participants in all three categories stressed that non-adherence to religious beliefs and practices among adolescents is a facilitator to engaging in risky sexual behaviors. This may be due to the absence of internal and social control among adolescents, who are easily influenced by the prevailing temptations in society, as well as their lack of an ethical reference to distinguish between healthy and unhealthy behaviors, especially regarding risky sexual practices. In addition, religiosity, the cognitive, affective, and behavioral relationships between individuals and their religion, can influence decision-making by contributing to the concept of personal identity and by standardizing certain values and beliefs [68]. In contrast, it was also stated that adolescents with strong religious affiliations and practices tend to delay or avoid early sexual intercourse before marriage as it is morally unacceptable, showing that religious belief can be a protective factor against the risk of STIs, as previously documented [10, 56, 60, 62]. Indeed, greater religiosity implies greater coherence between religious values and behaviors, as well as greater resistance to modifying existing values and behaviors [68]. Religiosity and spirituality can also be a positive buffer and serve as an internal and social control, reducing or even restricting certain behaviors and helping adolescents make positive choices, such as avoiding premarital sexual activity [62]. Furthermore, those who abide by religion tend to establish harmonious relationships with friends who are similar to themselves and less sexually permissive, as highlighted in this study [58]. Adolescent who practice religion also tend to have more opportunities to communicate with adults who could advise them against unhealthy sexual behaviors in the context of religious organizations.

## Intervention suggestions

According to the participants, sexual health interventions should be implemented in a socio-ecological perspective, taking into consideration several influencing factors targeting adolescents and their environment. This approach may reduce STI acquisition and improve adolescent sexual health in general, as recommended previously [32].

As suggested before [36, 49, 50, 52], many participants in all three groups believed that effective interventions should include strengthening adolescent knowledge about sexuality, religious and moral beliefs, and adequate social and life skills. In addition, interventions must involve parents, including providing them with adequate and correct information on sexuality, developing their parenting and communication skills, and promoting effective parental monitoring. Interventions that include improving adolescent knowledge and skills and parental involvement have been found to be more effective in reducing risky sexual behavior, STIs, and pregnancy among students, as previously elucidated [69]. Furthermore, consistent with other studies [49], adolescents suggested that sexual health interventions delivered by prosocial and active peer educators would positively impact adolescent sexual health education because they believed that teens communicate and influence each other more than other groups. Favorable and supportive environments in which adolescents can develop social, behavioral, and communication skills, as previously shown with peer-led education programs in HIV prevention, appear to be effective ways to improve knowledge and attitude [37, 70].

As previously reported [31, 49, 50, 60, 61], most participants in all three categories suggested that the school could highly contribute to improving adolescent sexual health and reducing the incidence of STIs by promoting sex education in the school curriculum or including it as a school subject. A number of adolescents and teachers stressed that the information must be delivered by well trained teachers who are given sufficient training time and access to modern technologies to enable them to communicate the necessary information and skills related to this field in an interactive and participatory manner. Having well-trained and equipped teachers would give adolescents the opportunity to interact and learn from positive role models, allowing them to adopt positive behaviors [60]. Likewise, many teachers and parents suggested that adolescents must be given more opportunities to participate in extracurricular activities and school clubs, as previously reported [37, 50, 60]. These activities provide important protective factors that give adolescents opportunities to develop their skills to reject harmful behaviors in addition to providing less idle time.

Other participants in all three FGDs suggested that sexual health intervention can be delivered at school via awareness campaigns in partnership with school clubs and civil society associations; they also stressed that involving students effectively in the organization of these campaigns and relying on healthcare providers and specialists can have a positive outcomes on the students’ sexual health, as previously reported [49]. Some parents suggested field visits to hospitals to allow students to witness cases of STIs in person [49]. Interventions that include active participation of adolescents have been shown to have positive impacts on adolescent risky sexual behaviors [34, 69] because active participation makes them more attentive and more concerned.

It was also suggested that media outlets can play a more positive role in promoting adolescents’ sexual health by limiting the display of movies and television series that contradict the national conservative norms and encourage risky sexual behaviors, a finding consistent with other studies [62, 64], and by taking responsibility to raise awareness among adolescents on all forms of STIs; not only HIV [10, 56, 57]. Accessible programs can be supervised by physicians, psychologists, and celebrities who have a positive influence on teenagers.

Parents and teens also perceived that government officials should have an important role regarding steps to stop unfaithfulness and sex outside marriage norms and also blocking pornographic sites. However, these steps require the involvement and mobilization of all political and civil actors, which is not an easy task to achieve due to the difficulty of this compatibility between all opposing political, intellectual, and religious orientations within the community. One study suggested that policymakers should adopt policies that reduce socio-economic inequalities, which would reduce undesirable consequences, such as prostitution and other risky sexual behaviors [56]. In the present study, parents stressed that the government should create youth centers and theaters and promote sport activities so that adolescents have ample chances to release energy, occupy their free time, develop their social and communication skills, and increase their sense of responsibilities, thus, improving their sexual behaviors. This finding, which is consistent with a previous study, showed that, instead of directly addressing the sexual risk of adolescents, parents seemed to prefer keeping their adolescents busy with activities [63].

Parents and teachers suggested that the Ministry of Health must raise adolescent awareness about sexuality and STIs through ongoing interventions at school, including conducting confidential STI screening campaigns among students, which is in agreement with previous recommendations [10]. Similarly, this was reported in a prior study [53] in which parents and teachers supported school-centered STI screening for adolescent girls. However, the issue of whether to disclose STI results to parents remains dependent on the social and legal context in which the intervention takes place.

## Limitations

This study has several limitations. First, only two schools in an urban area of Morocco were included. Consequently, our results are probably not generalizable to a larger population. Nevertheless, the generalization of the results was not our aim; as much as to explore the perceived ecological risks and protective factors of risky sexual behavior among adolescents. Due to the qualitative nature of the research, it was not possible to define which influencing factors were most important regarding sexual risk behaviors in adolescents. Second, because this was a qualitative study, care must be taken not to infer causality between perceived factors and sexual behavior in adolescents. Additionally, it would have also been useful if there had been key informant interviews to collect information from individual experts; however, such methods would require careful selection of subjects to get input from the most knowledgeable people and require meeting with many people to produce results that can be generalized, which is difficult in our context due to the scarcity of experts concerned with adolescent sexual health.

Despite these limitations, this first qualitative study in Morocco on risky sexual behavior in adolescents allowed us to gain a holistic understanding of influencing factors. A number of interventions to improve sexual health and reduce STIs among adolescents were suggested by participants. Furthermore, our theoretical framework, triangulation, and the large number of participants and the rigorous methodology applied allowed a more complete overview of this subject.

## Conclusions

The influencing factors identified in this study belong to different ecological levels within an adolescent’s life. It has been observed that socio-cultural and personal barriers in Morocco led to a lack of knowledge, an increase of sensation-seeking behaviors, lack of parental communication about sexuality, and lack of sex education within the school, which promote risky sexual behaviors in adolescents. In addition, due to globalization and access to technology and media, social norms have changed, leading to normalization of risky sexual behaviors. On the other hand, within individual influences, we found that awareness and fear of STIs, pregnancy, and shame can protect against risky sexual behaviors. Similarly, among social-environmental influences, maternal communication, prosocial peers, extracurricular activities, and religiosity were considered protective. Participants also suggested that interventions based on a multicomponent approach can be effective in delaying adolescent sexual activity and reducing STI acquisition.

## Recommendations

Based on the study findings and conclusions, a number of recommendations emerged, as listed below:

- Parents, teachers, and adolescents must join efforts in developing and implementing effective interventions that address and reduce identified facilitators of risky behavior, particularly working to lift socio-cultural and personal barriers that prevent effective communication between adolescents and adults and providing adequate sex education for both parents and adolescents.
- Schools can be an effective setting for reducing risky sexual behaviors in adolescents by providing sex education in the school curriculum, perhaps through reinforcement of religious beliefs related to sexual behavior and teaching communication and life skills.
- The engagement of adolescents in extracurricular activities can improve confidence and help teens make healthy decisions.
- Participants stressed that policymakers must contribute to reduce these risks among adolescents by creating cultural and sportive facilities that enable adolescents to make a good use of their leisure time and develop their communication and life skills.
- Media should also contribute by broadcasting messages to teens not to engage in risky sexual practices and by blocking pornographic sites.

## Data Availability

The datasets underlying the results are not accessible to the public since the participants did not give their consent for the public sharing of their information. However, a summary of transcribed interviews is available from the corresponding author upon reasonable request. Discussion guides used in this study are also available upon request.

## Abbreviations

AIDS: Acquired immune deficiency syndrome
FGDs: Focus group discussions
HIV: Human immunodeficiency virus
STIs: Sexually transmitted infections
